# Development and validation of accelerated failure time model for cause-specific survival and prognostication of oral squamous cell carcinoma: SEER data analysis

**DOI:** 10.1371/journal.pone.0309214

**Published:** 2024-08-26

**Authors:** Phillip Awodutire, Michael Kattan, Oladimeji Adeniyi Akadiri

**Affiliations:** 1 Department of Quantitative Health Sciences, Cleveland Clinic, Cleveland, Ohio, United States of America; 2 Department of Oral and Maxillofacial Surgery, University of Port Harcourt, Choba, Nigeria; Tabriz University of Medical Sciences, ISLAMIC REPUBLIC OF IRAN

## Abstract

**Background:**

Oral Squamous Cell Carcinoma is the most prevalent malignancies affecting the oral cavity. Despite progress in studies and treatment options its outlook remains grim with survival prospects greatly affected by demographic and clinical factors. Precisely predicting survival rates and prognosis plays a role in making treatment choices for the best achievable overall health outcomes.

**Objective:**

To develop and validate an accelerated failure time model as a predictive model for cause-specific survival and prognosis of Oral Squamous Cell Carcinoma patients and compare its results to the traditional Cox proportional hazard model.

**Method:**

We screened Oral cancer patients diagnosed with Squamous Cell Carcinoma from the Surveillance Epidemiology and End Results (SEER) database between 2010 and 2020. An accelerated failure time model using the Type I generalized half logistic distribution was used to determine independent prognostic factors affecting the survival time of patients with oral squamous carcinoma. In addition, accelerated factors were estimated to assess how some variables influence the survival times of the patients. We used the Akaike Information Criterion, Bayesian Information Criterion to evaluate the model fit, the area under the curve for discriminability, Concordance Index (C-index) and Root Mean Square Error and calibration curve for predictability, to compare the type I generalized half logistic survival model to other common classical survival models. All tests are conducted at a 0.05 level of significance.

**Results:**

The accelerated failure time models demonstrated superior effectiveness in modeling (fit and predictive accuracy) the cause-specific survival (CSS) of oral squamous cell carcinoma compared to the Cox model. Among the accelerated failure time models considered, the Type I generalized half logistic distribution exhibited the most robust model fit, as evidenced by the lowest Akaike Information Criterion (AIC = 27370) and Bayesian Information Criterion (BIC = 27415) values. This outperformed other parametric models and the Cox Model (AIC = 47019, BIC = 47177). The TIGHLD displayed an AUC of 0.642 for discrimination, surpassing the Cox model (AUC = 0.544). In terms of predictive accuracy, the model achieved the highest concordance index (C-index = 0.780) and the lowest root mean square error (RMSE = 1.209), a notable performance over the Cox model (C-index = 0.336, RMSE = 6.482). All variables under consideration in this study demonstrated significance at the 0.05 level for CSS, except for race and the time span from diagnosis to treatment, in the TIGHLD AFT model. However, differences emerged regarding the significant variations in survival times among subgroups. Finally, the results derived from the model revealed that all significant variables except chemotherapy, all TNM stages and patients with Grade II and III tumor presentations contributed to the deceleration of time to cause-specific deaths.

**Conclusions:**

The accelerated failure time model provides a relatively accurate method to predict the prognosis of oral squamous cell carcinoma patients and is recommended over the Cox PH model for its superior predictive capabilities. This study also underscores the importance of using advanced statistical models to improve survival predictions and outcomes for cancer patients.

## Introduction

Oral squamous cell carcinoma (ORSCC) is the most prevalent form of oral cancer, accounting for 90% of cases in the oral cavity [1–3]. It represents 2–3% of all newly diagnosed malignancies in the United States, ranking as the 8th and 14th most common malignancy among men and women, respectively [4,5]. Globally, ORSCC is the 13th most common malignancy, with an estimated incidence of 377,713 new cases and 177,757 deaths in 2020, according to WHO [6]. Multiple risk factors are known, including smoking and alcohol intake, whose synergistic effect heightens the risk by 100-fold. Other associated factors are infection, sun exposure, chronic irritability, poor oral hygiene, malnutrition, immunosuppression, and genetic disorders [7]. Some cases are preceded by identifiable premalignant lesions or carcinoma in situ. Hence, some researchers argue that dental professionals should screen all at-risk patients to detect oral cancer at an early stage [8,9]. Similarly, the American Cancer Society (ACS) recommends including an oral cavity cancer examination in routine health check-ups, especially for individuals of certain age groups [10]. Despite advancements in surgical and medical management, treatment outcomes for ORSCC remain poor [11]. The 5-year survival rate for early-stage ORSCC is approximately 90%, dropping significantly to 30% for late-stage diagnoses [12]. The disease outcome involves a multiplex interaction of clinical, pathological, molecular, and miscellaneous factors [13,14]. Hence, predicting the prognosis of ORSCC remains a challenging task [15] but a key step for treatment planning. Researchers have developed numerous tools to predict survival rates for ORSCC patients. This includes identifying novel biomarkers for prognosis prediction and therapy monitoring and assessing traditional clinical and pathological variables such as age, gender, race, clinical stage, tumor location, tumor grade, and applied treatment modality. [16–22].

Generally, prognostic models integrating multiple clinical attributes have been developed in different oncological practices to achieve more precise clinical outcome predictions. Jhadav et al. [14] created a multifactorial instrument for predicting the survival of ORSCC patients by ascribing arbitrary scores to several clinicopathologic parameters. No statistical basis was established, and the instrument was not validated in any study. On a different plain, nomograms, statistical tools visualizing complex models that utilize clinical characteristics to predict individual patient outcomes, are widely used to evaluate the prognosis of various cancers, including cervical, breast, and lung [23–27]. Researchers have also attempted to develop prognostic nomograms for ORSCC [28–30]. These are limited by their small sample sizes, homogeneity of the study population, and restriction to post-resection cases; hence, their generalizability is in question, necessitating further exploration and validation through larger-scale studies. Furthermore, these studies, as well as many other survival analytics studies, have employed the traditional Cox proportional hazard (Cox PH) model approach for predicting ORSCC outcomes; this method has limitations, one of which is the proportional hazard assumptions, meaning that the relative hazard remains constant over time despite different predictor or covariate levels. This is an unlikely reality in a disease prognosis with a wide spectrum of co-determinants such as ORSCC.

The accelerated failure time (AFT) model has been an alternative to the Cox model in survival analysis. It directly models the survival times to the prognostic factors, making it easy to compute and interpret. This model assumes that the survival time follows a particular probability distribution function. Furthermore, it assumes that the effects of the factors/covariates can decelerate or accelerate survival times. This implies that AFT draws conclusions on the event time, making it easy to interpret and infer. This approach has been found to have great predictive accuracy and can control overfitting due to its flexibility from model parameters. According to Aelen [31], using the Accelerated Failure Time model approach, which generates efficient and consistent estimates in predictive modeling, has had limited application in medical time-to-event analyses. One notable metric of the AFT model is the Acceleration Factor (AF), which compares the survival time between two variables. It tells how covariates accelerate or decelerate the time to the event of interest. Some common probability distribution functions used as the baseline for AFT models include Weibull, lognormal, exponential, log-logistic, etc. These AFT models have been explored in several studies and were found to be preferred to the Cox model [32–36].

The TIGHLD survival model was derived by Awodutire et al. [37] and explored by assessing factors contributing to breast cancer patients’ survival in Nigeria [38]. The model’s results were compared to that of classical models, showing superior performance.

This study aims to explore the TIGHLD to construct a new Accelerated Failure Time (AFT) model for predicting the prognosis and CSS of ORSCC patients based on clinical and pathological characteristics. This AFT model will enable clinicians to predict patient prognosis and assist in treatment planning accurately. Also, this research aims to assess and compare the predictive accuracy of the new AFT model, built on advanced parametric modeling, to some existing classical AFT models and the traditional Cox proportional hazard (Cox PH) model by employing diverse diagnostic approaches to achieve this objective. Furthermore, the Accelerator Factor (AF) would be derived from knowing how some variables accelerate or decelerate CSS in patients with ORSCC.

## Methods

### Study design

This is a retrospective analysis of extracted information on oral cancer patients with histological diagnosis of squamous cell carcinoma between 2010 and 2020 across 17 registries from the Surveillance Epidemiology and End Results (SEER) database. Patients with incomplete or missing data were excluded, while all included patients were randomly split 70–30 into the training and validation cohorts, respectively.

### Data collection and clarifications

Information collected from the database included independent variables such as the patient’s demographic characteristics (Age, gender, Race), tumor primary site, the total number of in situ malignant tumors, the month of diagnosis to treatment, clinical stage of the tumor, histological Grade of the tumor, and treatment modality. Also, outcome/response variables such as death or alive (binary outcome) and Cause-Specific Interval (CSI: i.e., survival time (in months) from diagnosis to death) were recorded. For demographic data, gender was binary (male or female). Age was subclassified using non-uniform intervals (05–19, 20–39, 40–59, 60–74, >75 years), and race was in four categories (White, Black, American Indian, and Asian). The tumor sites were buccal mucosa (BM), the floor of the mouth (FoM), Gum(G), Lip (L), other parts of the mouth (OpM), palate(P), and tongue anterior (TA). Treatment modalities were aggregated into three groups, namely: No Intervention, Chemotherapy only (monotherapy), radiation+surgery (bimodal therapy), and radiation+surgery+chemotherapy (trimodal therapy). Histological grades were classified as well differentiated (G1), moderately differentiated (G2), poorly differentiated (G3), and undifferentiated (G4).

### Ethical consideration

Since the SEER database is publicly accessible, obtaining informed patient consent for this study was unnecessary, identification parameters were not retrieved to preserve anonymity. The study was considered exempt from review by the Ethics Committee of the Cleveland Clinic.

### Accelerated failure time model

Let S_0_(t_i_) denote the baseline survival function, γ the vector of regression coefficients, x_i_ the covariates, then under the AFT model the survival function S(t_i_/x_i_) of the failure time T is

$$S(t_{i}|x_{i},\gamma_{i})=S_{0}(t_{i}\cdot exp(x_{i}\gamma_{i}))$$

with probability density function

$$f(t_{i}|x_{i},\gamma_{i})=exp(x_{i}\gamma_{i})f_{0}(t_{i}\cdot exp(x_{i}\gamma_{i}))$$

and hazard function

$$h(t_{i}|x_{i},\gamma_{i})=exp(x_{i}\gamma_{i})h_{0}(t_{i}\cdot exp(x_{i}\gamma_{i}))$$

where S_0_(.), f_0_(.), and h_0_(.) are the baseline survival, probability density, and hazard functions, respectively, and $x_{i}\gamma_{i}=x_{1}\gamma_{1}+x_{2}\gamma_{2}+x_{3}\gamma_{3}+\ldots$

*The type I generalized half logistic distribution has a distribution function as in equation* [Eq 4]

$$f_{0}(t,\sigma ,b)=\frac{b2^{b}e^{\frac{t}{\sigma }}}{\sigma {\left(1+e^{\frac{t}{\sigma }}\right)}^{b+1}}$$

*with the cumulative distribution function as*

$$F_{0}(t,\sigma ,b)=1-\frac{2^{b}}{{\left(1+e^{\frac{t}{\sigma }}\right)}^{b}}$$

*the survival function as*

$$s_{0}(t,\sigma ,b)=\frac{2^{b}}{{\left(1+e^{\frac{t}{\sigma }}\right)}^{b}}$$

*where t*>*0*, *σ*,*b*>0. *b and σ is the shape and scale parameter respectively*.

### Statistical analysis

The entire SEER data that met inclusion criteria from the 17 registries were merged and randomly split 70–30 into training and validation data sets respectively, which were large enough for the construction of the new model (training data cohort) and for evaluation of model performance within the treatment subgroups (validation data cohort). The statistical analysis was executed in three phases: 1. a general description of baseline characteristics and summary statistics; 2. construction and validation of a new AFT model for survival analysis of ORSCC and determination of the accelerator factors (AFs); 3. determination and comparison of the predictivity and discriminability of the new AFT model in comparison to three classical AFT models and the traditional Cox proportional hazard (Cox PH) model.

### General description of baseline characteristics in summary statistics

Patient and disease characteristics of the training data were summarized using the median and interquartile range for continuous variables and the number and percentage for categorical variables. Fisher’s exact test and the Wilcoxon rank-sum test were used to test for univariable differences according to censoring status for categorical and continuous variables, respectively. The summary statistics were utilized to describe the baseline characteristics of patients along with the study outcome, where continuous variables were represented as the median and frequency for the categorical variables. The Kaplan-Meier method was employed to compare survival rates within groups in the categorical variables. The Schoenfield residual test was first conducted to assess the hazard proportionality assumption of the variables.

### Construction and validation of the new AFT model and determination of accelerator factors (AF)

Patients in the training cohort were retrospectively analyzed to construct the accelerated failure time model using the Type I generalized half logistic distribution (TIGHLD). The model was internally validated with the validation data set. Follow-up was administratively censored for four years. The TIGHLD AFT model was applied to assess the variables’ relative contribution to the ORSCC patients’ survival. Furthermore, AF determination followed these stated criteria: If AF > 1, exposure benefits survival; if AF < 1, exposure is harmful to survival; and if AF = 1, there is no effect from exposure. An AF greater than 1 means that the predictor variable is associated with a longer survival time when compared to the reference variable, and an AF less than 1 means that the predictor variable is associated with a shorter survival time when compared to the reference variable.

### Comparison of the predictivity and discriminability of the new AFT model

The model fit of the newly developed prediction model to the dataset was compared with that of two classical AFT models, i.e., the Lognormal and Weibull AFT models and the traditional Cox proportional hazard (Cox PH) model, by a rigorous process using the Akaike Information Criterion (AIC) and Bayesian Information Criterion (BIC) whereby the model with the lowest values is considered the best fit. Furthermore, the area under the curves (AUC) was used to evaluate the discrimination performance of the prediction models in the internal validation data. The root mean square error (RMSE) and Concordance Index (C-index) measures were used to quantify how well the derived model’s predicted probabilities align with the actual outcomes regarding survival times. A higher concordance index and the lowest RMSE indicate better predictive accuracy. A calibration plot was used further to explore the accuracy of the prediction model. The Acceleration Factor for the categorical variables was estimated to assess how much the variables accelerate or decelerate the time to death. These statistical analyses were conducted using R version 4.3.1 software (The R Foundation for Statistical Computing, Vienna, Austria; www.r-project.org). All the tests were done with a p-value of less than 0.05, considered statistically significant.

## Results

**Fig 1:**
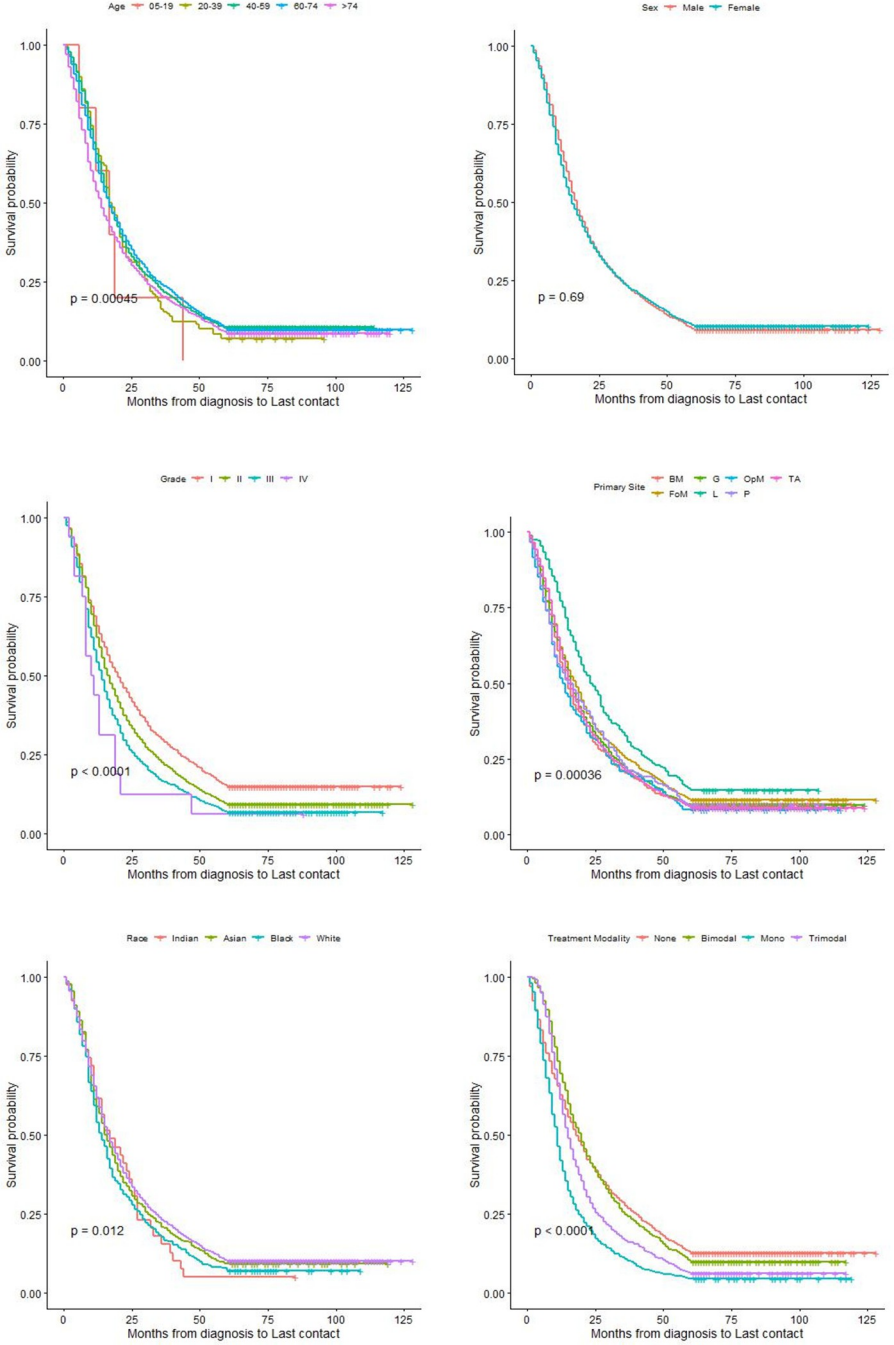
Kaplan Meier plot of ORSCC survival stratified by Categorical Covariates. (A) Kaplan Meier plot of ORSCC survival stratified by Age. (B) Kaplan Meier plot of ORSCC survival stratified by Grade. (C) Kaplan Meier plot of ORSCC survival stratified by Race. (D) Kaplan Meier plot of ORSCC survival stratified by Sex. (E) Kaplan Meier plot of ORSCC survival stratified by Primary Site. (F) Kaplan Meier plot of ORSCC survival stratified by Treatment Modality.

There were 5,084 patients with ORSCC from the SEER database included in this study, of whom 4,588 (90%) had the observed event of interest(death), and 496(10%) were censored, as in Table 1. When split, 3,561 patients were in the training cohort and 1,523 patients in the validation cohort. Most ORSCC occurs on the tongue anterior site (TA), and the Grade II tumor was most prevalent at diagnosis and treatment. Only 54% of the patients had at least an intervention in the form of monotherapy by Chemotherapy alone, bimodal therapy in the form of radiation and surgery, or trimodal therapy involving surgery, radiation, and Chemotherapy. Table 1 shows the frequency distribution of the entire data set, the training, and the validation data cohorts, which shows a similar distribution in sex, age, tumor grade, treatment modality, and CSI (cause-specific interval). This was further described using the Kaplan-Meier plots in Fig 1A–1F.

**Table 1:** Descriptive statistics of the patients’ characteristics.

Characteristics	Total		Train			Test	
	N = 5,084	N = 339^*0*^	N = 3222^*1*^	p-value	N = 157^*0*^	N = 1366^*1*^	p-value
Race				0.094			0.2
American Indian/Alaska Native	39 (0.8%)	1 (0.3%)	25 (0.8%)		1 (0.6%)	12 (0.9%)	
Asian or Pacific Islander	389 (7.7%)	18 (5.3%)	252 (7.8%)		18 (11%)	101 (7.4%)	
Black	407 (8.0%)	20 (5.9%)	266 (8.3%)		9 (5.7%)	112 (8.2%)	
White	4,249 (84%)	300 (88%)	2,679 (83%)		129 (82%)	1,141 (84%)	
Sex				0.11			>0.9
Male	3,143 (62%)	195 (58%)	1,995 (62%)		98 (62%)	855 (63%)	
Female	1,941 (38%)	144 (42%)	1,227 (38%)		59 (38%)	511 (37%)	
Age				0.13			>0.9
05–19	5 (<0.1%)	0 (0%)	3 (<0.1%)		0 (0%)	2 (0.1%)	
20–39	128 (2.5%)	6 (1.8%)	89 (2.8%)		3 (1.9%)	30 (2.2%)	
40–59	1,459 (29%)	114 (34%)	903 (28%)		42 (27%)	400 (29%)	
60–74	1,929 (38%)	130 (38%)	1,215 (38%)		63 (40%)	521 (38%)	
>75	1,563 (31%)	89 (26%)	1,012 (31%)		49 (31%)	413 (30%)	
Grade				<0.001			0.022
Well differentiated; Grade I	937 (18%)	96 (28%)	560 (17%)		43 (27%)	238 (17%)	
Moderately differentiated; Grade II	2,979 (59%)	189 (56%)	1,890 (59%)		87 (55%)	813 (60%)	
Poorly differentiated; Grade III	1,152 (23%)	53 (16%)	761 (24%)		27 (17%)	311 (23%)	
Undifferentiated; anaplastic; Grade IV	16 (0.3%)	1 (0.3%)	11 (0.3%)		0 (0%)	4 (0.3%)	
Site				0.2			0.7
Buccal Mucosa	456 (9.0%)	31 (9.1%)	282 (8.8%)		14 (8.9%)	129 (9.4%)	
Floor Of Mouth	820 (16%)	62 (18%)	495 (15%)		32 (20%)	231 (17%)	
Gum	989 (19%)	64 (19%)	628 (19%)		31 (20%)	266 (19%)	
Lip	196 (3.9%)	20 (5.9%)	113 (3.5%)		9 (5.7%)	54 (4.0%)	
Mouth Other	215 (4.2%)	11 (3.2%)	130 (4.0%)		7 (4.5%)	67 (4.9%)	
Palate Excluding Soft and Uvula	174 (3.4%)	10 (2.9%)	109 (3.4%)		7 (4.5%)	48 (3.5%)	
Tongue Anterior	2,234 (44%)	141 (42%)	1,465 (45%)		57 (36%)	571 (42%)	
Treatment Modality				<0.001			0.003
No Treatment administered	2,338 (46%)	211 (62%)	1,449 (45%)		87 (55%)	591 (43%)	
radiation and/or cancer-directed surgery only	998 (20%)	63 (19%)	622 (19%)		36 (23%)	277 (20%)	
Chemotherapy Only	702 (14%)	20 (5.9%)	453 (14%)		13 (8.3%)	216 (16%)	
radiation and/or cancer-directed surgery and Chemotherapy	1,046 (21%)	45 (13%)	698 (22%)		21 (13%)	282 (21%)	
Survival Time in months	16 (9, 33)	76 (68, 90)	15 (8, 26)	<0.001	76 (68, 92)	14 (8, 26)	<0.001
Months from diagnosis to treatment	1.00 (0.00, 2.00)	1.00 (0.00, 2.00)	1.00 (1.00, 2.00)	<0.001	1.00 (0.00, 2.00)	1.00 (1.00, 2.00)	0.024
Total number of in situ malignant tumors	1.00 (1.00, 2.00)	2.00 (1.00, 3.00)	1.00 (1.00, 2.00)	<0.001	2.00 (1.00, 3.00)	1.00 (1.00, 2.00)	<0.001
AJC_stage				<0.001			<0.001
I	995(19%)	137 (40%)	568 (18%)		56 (36%)	234 (17%)	
II	692(14%)	60 (18%)	421 (13%)		33 (21%)	178 (13%)	
III	751(15%)	51 (15%)	498 (15%)		16 (10%)	186 (14%)	
IV	2346(46%)	60 (18%)	1,562 (48%)		41 (26%)	683 (50%)	
V	300(6%)	31 (9.1%)	173 (5.4%)		11 (7.0%)	85 (6.2%)	

*0 = censored *1 = Uncensored.

Table 2 shows the output of the Schoenfield residual test conducted to assess the proportional hazard assumption of the predictive variables. The assumption is not fulfilled for sex (p = 0.024), age (p = 0.000), Grade (p = 0.042), Tumor Site (p = 0.009), Treatment (p = 0.000), and TNM stage (p = <0.001). This implies that the Cox model is not suitable for modeling this dataset.

**Table 2:** Schoenfield test for hazard proportionality.

Variables	chisq	df	p-value
Race	3.490	3	0.322
Sex	5.110	1	0.024*
Age	27.740	4	<0.001*
Grade	5.860	3	0.119
Primary Site	17.000	6	0.009*
Treatment	59.570	3	<0.001*
TNM stage	21.360	1	<0.001*

*chisq = Chi square statistics *df = degrees of freedom.

As an alternative to the Cox model, the AFT model was applied. The main assumption for any baseline distribution for the AFT model is that the survival time follows the probability distribution. Three probability distributions (Weibull, Lognormal, and Type I generalized half logistic distribution (TIGHLD)) were considered in developing the AFT model of survival time of the OSRCC patients. Table 3 shows that the TIGHLD AFT model best fits the dataset due to its lowest values for the AIC (29870) and the BIC (29873), followed closely by the lognormal distribution. This indicates that the survival time distribution of the dataset follows the TIGHLD better than the other two probability distributions considered.

**Table 3:** Probability distribution fit of the survival time of OSRSS patient.

Distribution	Parameters	loglik	AIC	BIC
Weibull	2	14970	29944	29947
Lognormal	2	14951	29904	29909
TIGHLD	2	14933	29870	29873

*loglik = loglikelihood value.

**Fig 2:**
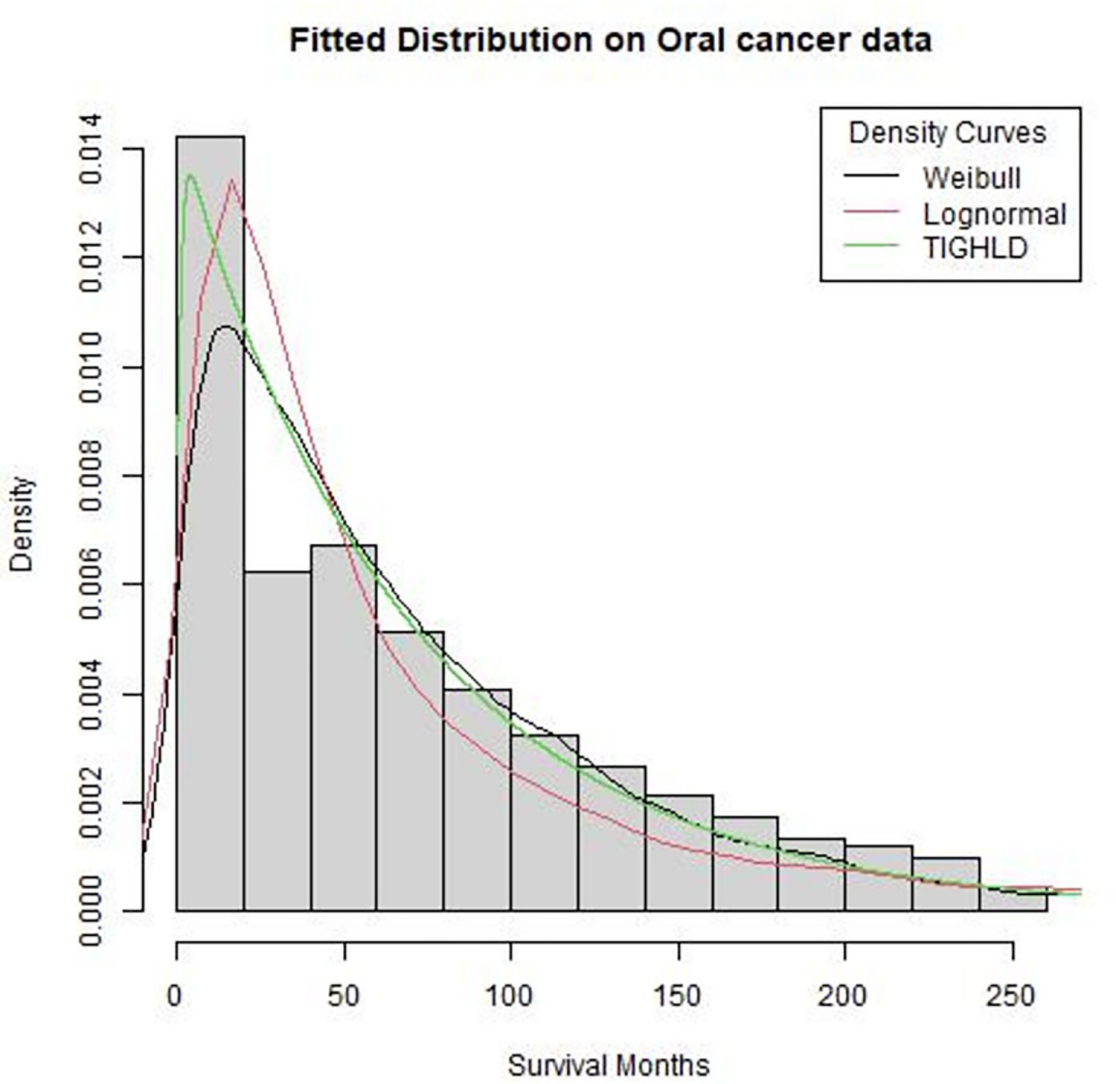
Probability distribution fit of the TIGHLD, lognormal and weibull distributiond to the OSRCC survival times.

Fig 2 further gives the calibration of the plots of the distributions on the survival time, which revealed how close the three distributions (i.e., TIGHLD, lognormal and Weibull) performed in fitting the survival time data. Therefore, the TIGHLD distribution is preferred as the baseline distribution for the AFT model for prognosis analysis of the OSRCC dataset.

Modeling the survival time on the intercept using the four survival models also revealed the TIGHLD AFT model as the best fit with the lowest AIC (27770) and BIC (27774), compared to the Cox PH model AIC (47698) and BIC (47700) in Table 4.

**Table 4:** Survival model fit with intercept of the CSS of ORSCC patient.

	Parameters	loglik	AIC	BIC
Weibull	3	13882	27770	27774
Lognormal	3	13650	27306	27310
TIGHLD	3	13646	27298	27302
Cox	1	23849	47698	47700

*loglik = loglikelihood value.

Considering the covariates, the AFT and Cox PH models were applied to the Cause-specific survival (CSS) of the ORSCC patients. The goodness of fit, discrimination, and predictability accuracy of these models are presented in Table 5. For validation, the model performed well in discrimination ability (AUC = 0.640) compared to the Cox model(AUC = 0.544). Furthermore, the TIGHLD AFT model performs better than the competitive models). To further establish the superiority in model fit of the TIGHLD AFT model, compared to the other models, the TIGHLD AFT model has the lowest AIC (27370) and BIC (27415). For predictability accuracy, the TIGHLD model has the highest C-Index (0.780) and lowest RMSE (1.2094) among the models, indicating better predictive accuracy in survival times.

**Table 5:** Survival model fit with prognostic factors of the CSS of ORSCC patient.

		Model Fit			Discrimination	
Model	Parameters	Loglik	AIC	BIC	AUC	RMSE	C-Index
Weibull	29	13709	27476	27521	0.640	1.4825	0.688
Lognormal	29	14114	28286	28331	0.631	1.3642	0.770
TIGHLD	29	13656	27370	27415	0.642	1.2094	0.780
Cox	23	23483	47019	47177	0.544	6.482	0.336

*loglik = loglikelihood value.

**Fig 3:**
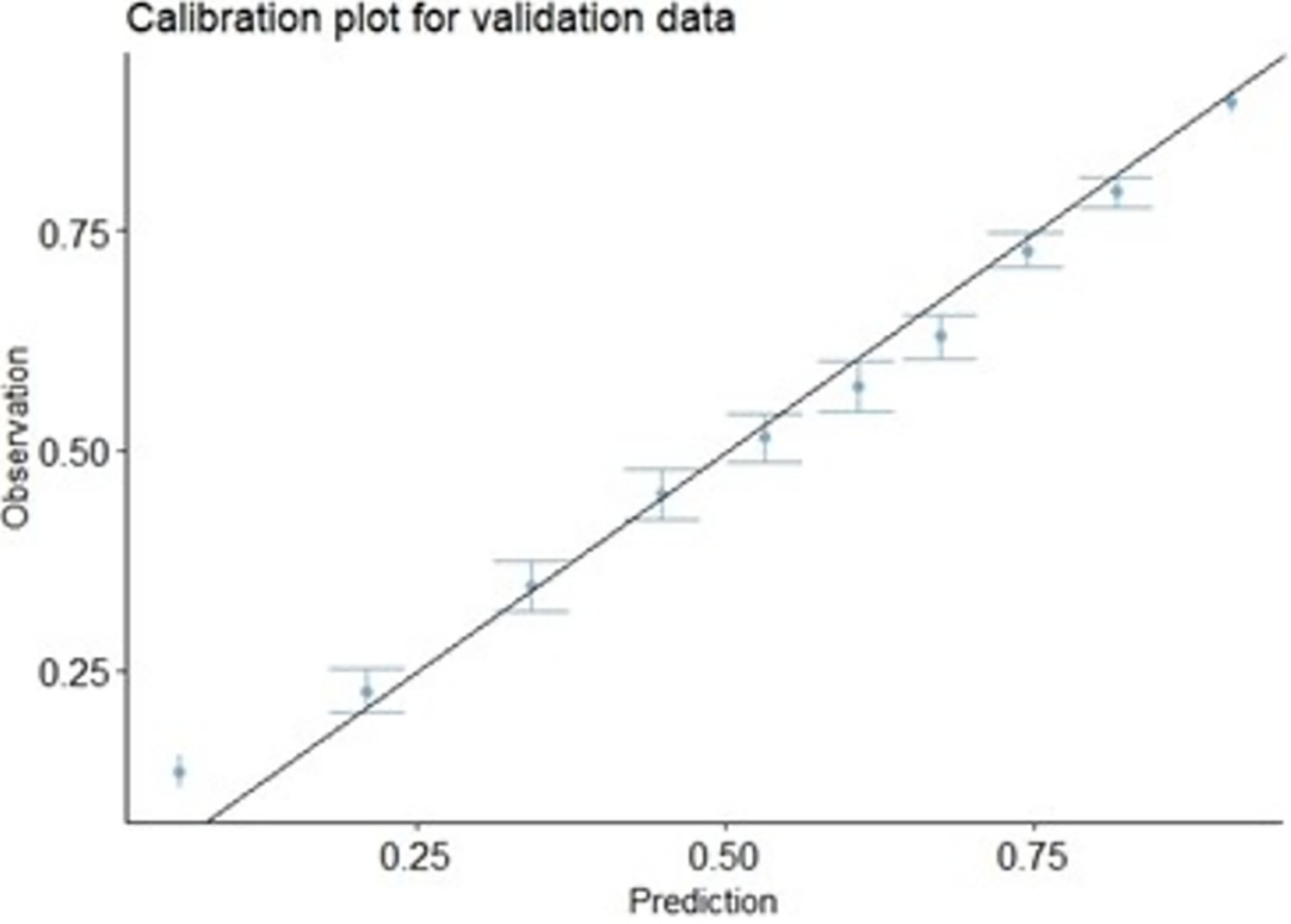


Fig 3 Calibration Plot of the TIGHLD AFT model cross-validated survival probability. Calibration is better when the points are closer to the 45° line.Based on the result in Table 5, the result of the prediction models using the TIGHLD AFT and the Cox PH for the ORSCC data were presented for comparison in Table 6. In Table 6 the result of the analysis using the TIGHLD AFT model shows that all factors are significant to CSS of ORSCC patients except for race, but within sub-categories, there are differences in their survival times, while the Cox PH model reveals that race and age categories were insignificant. For the TIGHLD AFT model, there is a statistical difference between the survival time among the categories. Furthermore, a negative value of the covariate’s estimates indicates the variables that decelerate the time to cause specific survival faster when compared to reference variable while increase in other factors accelerate it. These negative covariates are chemotherapy, all the TNM stages, gender, and tumor grade II and III, inidicating factors that decelerate survival. Fig 3 shows the calibration plot of the actual and predicted survival probability using the TIGHLD AFT model. It revealed the points close to the diagonal line, indicating a good calibration.

**Table 6:** TIGHLD AFT model to predict the CSS of ORSCC patients.

Variable Estimates		AF	p-value	Estimate	OR	p-value
**Age** 05–19*	-	-	-	-	-	-
20–39	0.957	2.604	0.007	-0.302	0.739	0.555
40–59	0.781	2.183	0.021	-0.258	0.772	0.607
60–74	0.725	2.064	0.032	-0.207	0.813	0.681
>75	0.572	1.771	0.091	0.049	1.051	0.922
**Race** American Indian/Alaska Native*	-	-	-	-	-	-
Asian or Pacific Islander	0.217	1.242	0.234	-0.196	0.822	0.346
Black	0.132	1.141	0.465	-0.156	0.856	0.452
White	0.066	1.068	0.701	-0.206	0.814	0.299
**Gender** Male*	-	-	-	-	-	-
Female	-0.080	0.923	0.016	0.077	1.080	0.041
**Tumor Site** Buccal Mucosa*	-	-	-	-	-	-
Floor Of Mouth	0.582	1.790	0.000	-0.106	0.899	0.157
Gum	0.436	1.546	0.000	-0.124	0.884	0.086
Lip	0.404	1.497	0.000	-0.224	0.799	0.042
Mouth Other	0.740	2.095	0.000	-0.018	0.983	0.867
Palate Excluding Soft and Uvula	0.471	1.601	0.000	-0.173	0.841	0.123
Tongue Anterior	0.341	1.406	0.000	0.019	1.019	0.769
**Grade** Well differentiated; Grade I*	-	-	-	-	-	-
Moderately differentiated; Grade II	-0.090	0.914	0.038	0.140	1.150	0.005
Poorly differentiated; Grade III	-0.153	0.858	0.003	0.285	1.330	0.000
Undifferentiated; anaplastic; Grade IV	0.971	2.641	0.128	0.399	1.491	0.173
**Total number of in situ malignant tumors for patient**	0.168	1.183	0.000	-0.024	0.977	0.077
**Time span (in months) from diagnosis to treatment**	0.004	1.004	0.721	-0.205	0.814	0.000
**Treatment Modality** No treatment Administered*	-	-	-	-	-	-
radiation and/or cancer-directed surgery only	0.188	1.207	0.000	-0.393	0.675	0.000
Chemotherapy Only	-0.185	0.831	0.000	0.132	1.141	0.025
radiation and/or cancer-directed surgery and Chemotherapy	0.240	1.271	0.000	-0.403	0.668	0.000
**TNM Stage**						
I	-	-	-	-	-	-
II	-0.368	0.692	0.000	0.371	5.679	0.000
III	-0.377	0.686	0.000	0.605	9.294	0.000
IV	-0.976	0.377	0.000	1.045	17.703	0.000
Unknown	-0.433	0.648	0.000	0.299	3.410	0.001

*Variable adopted as reference to which other variables are tested in the subgroup. *AF = Acceleration Factor *OR = Odd Ratio.

## Discussion

The news of a cancer diagnosis is always a traumatic announcement to cancer patients and their loved ones. It comes with much anxiety, doubts, fear of the unknown, and emotional conflicts between good wishes and bitter realities. So, understanding what is to be expected can help patients with lifestyle changes, coping with the impeded quality of life, making decisions on treatment options, and managing finances [14]. This understanding cannot come from diagnosis without prognosis. Hence, providing prognostic information to patients and relatives has become integral to oncological care. The prognostic information often delivered to patients diagnosed with ORSCC in the clinical settings is mostly based on the documented AJCC information, which is based mainly on TNM staging of the carcinoma based on the Cox proportional hazard model.

In contrast, many covariates in individual patients might change the outcome from one patient to another despite having the same TNM staging. Many other multivariable survival analyses have been published, but they are often derived from small sample sizes and usually not cause-specific but mere probabilities. To the best of these authors’ knowledge, this study provides the first CSS predictive model for ORSCC based on advanced parametric modeling of Accelerated Failure Time (AFT). Previous studies have shown that the AFT model’s parametric approach performs better in model estimation and predictive accuracy than the semiparametric (Cox model) approach. However, it is highly underused in medical research [31]. Furthermore, compared with traditional time-to-event models (e.g., Cox proportional hazard (Cox PH) model), AFT is sufficiently flexible to use in variables that fail to meet the proportional hazard assumption and very fit to model virtually any nonlinear relationship. Furthermore, the AFT model helps to assess how variables accelerate or decelerate survival time when compared to each category of patients within a variable.

This study included 5,084 patients with cause specific ORSCC from the SEER database. We had the training cohort (3,561) and internal validation cohort (1,523) from this population. The TIGHLD was considered a predictive model for ORCSS patients. The prognostic factors considered were race, age, sex, tumor grade, primary tumor site, pathological Grade, time span (in months) from diagnosis to treatment, the total number of in situ malignant tumors, TNM stage and treatment modality. The AFT model derived from the training cohort was then internally validated in the validation cohorts. The metrics showed that the TIGHLD AFT model performed best compared to the commonly used Cox PH and other competing AFT models through rigorous statistical processes. We infer from these findings that the TIGHLD AFT would be a handy clinical tool for CSS prediction, implying that it can distinguish actual cancer death from fatality due to co-morbidity or other causes different from carcinoma.

In comparison, the TIGHLD AFT and Cox PH model signified that race and time span (in months) from diagnosis to treatment were not a significant covariate for determining outcome among the independent variables integrated. Still, in terms of significant differences among sub-categories, the results differ. This is due to the ability of the AFT model to robustly detect significant variables, which in turn aids inference when compared to the Cox model. The TIGHLD AFT model further exemplified the relative contributions of the covariates (independent factors) in terms of their accelerative and decelerative potential for the outcome event. The results showed that all sub-categories accelerate survival times (AF > 1) except chemotherapy, TNM stages, gender and Grade II and III tumor category. This is extremely useful information for the clinician when evaluating disease prognosis and treatment planning. When externally validated, we believe the new AFT model developed in this study would be a robust instrument for developing a prognostic nomogram for ORSCC. Compared with previous attempts at developing such nomograms [29–30], the AFT is derived from a more extensive database and more rigorously assessed in comparison with existing models and has proven in this study to be better than the traditional Cox PH model that has been generously used in oral cancer research. Consistent with prior research works [25], our findings affirmed that a lower TNM stage, younger age, lower tumor grade, willingness to undergo surgery, and being female were indicative of better prognostic outcomes when compared to the control group. Furthermore, the chosen treatment approach significantly impacted CSS. The tumor’s primary site also held considerable sway over the prognosis. Lip tumors showed superior outcomes to tumors in other locations; this finding corroborates previous reports [13,14]. The same observation goes for the age. However, there is no significant difference between the survival times of the male and the female patients.

An identifiable downside to the newly developed model is that it does not consider the impact of molecular biomarkers. However, considering that biomarkers are not routinely assessed in clinical practice, this deficiency is unlikely to affect the clinical usefulness of this model. We, therefore, recommend this advanced parametric TIGHLD AFT model for survival analysis in the oncological care of oral squamous cell carcinoma to enhance decision-making and prognostic advisory to cancer patients and their relatives.

## Conclusion

Through this research work, an Accelerated Failure Time (AFT) model using the Type I generalized half logistic distribution (TIGHLD) has been developed and validated to predict survival time and prognostic factors of disease-specific mortality of ORSCC. The model endured the rigor of detailed statistical comparison with other classical models. Due to its flexibility and more accurate parameter estimations, the TIGHLD proved to be a better alternative to traditional Cox PH and AFT models and the best fit for oral squamous cell carcinoma.

## Supporting information

S1 Checklist (DOCX)
